# Exploring the Ocular Absorption Pathway of Fasudil Hydrochloride towards Developing a Nanoparticulate Formulation with Improved Performance

**DOI:** 10.3390/pharmaceutics16010112

**Published:** 2024-01-15

**Authors:** Barzan Osi, Ali A. Al-Kinani, Zinah K. Al-Qaysi, Mouhamad Khoder, Raid G. Alany

**Affiliations:** 1 Drug Discovery, Delivery and Patient Care (DDDPC) Theme, School of Life Sciences, Pharmacy and Chemistry, Kingston University London, London KT1 2EE, UK; a.alkinani@kingston.ac.uk (A.A.A.-K.); k1658781@kingston.ac.uk (Z.K.A.-Q.); m.khoder@kingston.ac.uk (M.K.); 2 School of Pharmacy, The University of Auckland, Auckland 1010, New Zealand

**Keywords:** ROCK, Fasudil hydrochloride, chitosan, nanoparticles, cornea absorption, ocular tolerability

## Abstract

Rho-kinase (ROCK) inhibitors represent a new category of anti-glaucoma medications. Among them, Fasudil hydrochloride, a selective ROCK inhibitor, has demonstrated promising outcomes in glaucoma treatment. It works by inhibiting the ROCK pathway, which plays a crucial role in regulating the trabecular meshwork and canal of Schlemm’s aqueous humor outflow. This study aims to investigate the ocular absorption pathway of Fasudil hydrochloride and, subsequently, develop a nanoparticle-based delivery system for enhanced corneal absorption. Employing the ionic gelation method and statistical experimental design, the factors influencing chitosan nanoparticle (Cs NP) characteristics and performance were explored. Fasudil in vitro release and ex vivo permeation studies were performed, and Cs NP ocular tolerability and cytotoxicity on human lens epithelial cells were evaluated. Permeation studies on excised bovine eyes revealed significantly higher Fasudil permeation through the sclera compared to the cornea (370.0 μg/cm^2^ vs. 96.8 μg/cm^2^, respectively). The nanoparticle size (144.0 ± 15.6 nm to 835.9 ± 23.4 nm) and entrapment efficiency range achieved (17.2% to 41.4%) were predominantly influenced by chitosan quantity. Cs NPs showed a substantial improvement in the permeation of Fasudil via the cornea, along with slower release compared to the Fasudil aqueous solution. The results from the Hen’s Egg Test Chorioallantoic Membrane (HET-CAM) and Bovine Corneal Opacity and Permeability (BCOP) tests indicated good conjunctival and corneal biocompatibility of the formulated chitosan nanoparticles, respectively. Lens epithelial cells displayed excellent tolerance to low concentrations of these nanoparticles (>94% cell viability). To the best of our knowledge, this is the first report on the ocular absorption pathway of topically applied Fasudil hydrochloride where the cornea has been identified as a potential barrier that could be overcome using Cs NPs.

## 1. Introduction

Glaucoma refers to a group of eye disorders with different clinical indications. Glaucoma causes progressive optic nerve neuropathy, leading to the loss of the visual field and, if not treated, blindness [1]. Generally, this occurs due to high pressure inside the eye, which can damage the optic nerve and retina by causing the loss of retinal ganglion cells [2]. However, it is also possible for glaucoma to occur in people with normal eye pressure, which typically ranges from 10 to 21 mmHg [3]. Among those, about 36 million people are blind, and an estimated 217 million experience significant visual impairment, ranging from moderate to severe [4]. In the UK, glaucoma impacts around 2% of individuals aged over 40 and increases to 10% among those older than 75, especially within the African-Caribbean population [5]. Treating ocular hypertension can be achieved by either decreasing the production of aqueous humor or increasing the outflow of aqueous humor.

**Figure 1:**
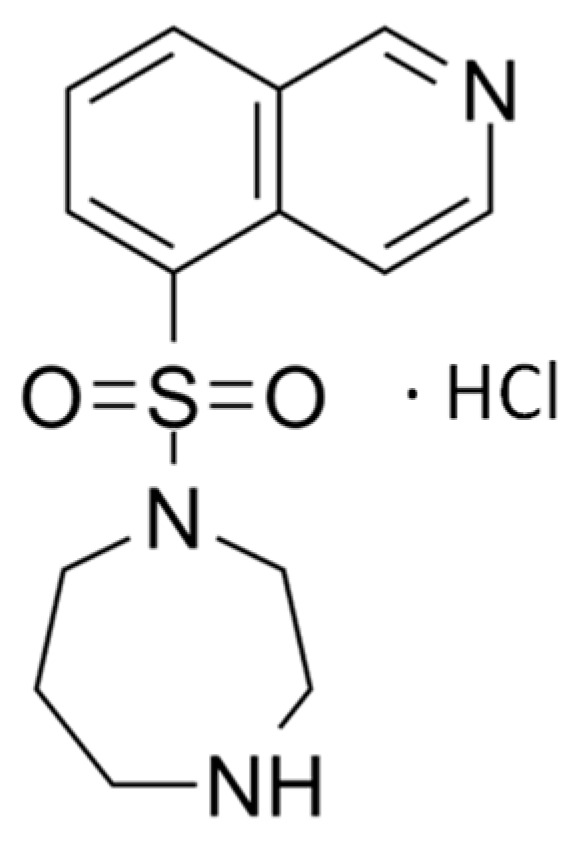
Chemical structure of Fasudil hydrochloride.

Rho-kinase (ROCK) inhibitors are a novel class of anti-glaucoma medicines [6,7]. Unlike other medicines, ROCK inhibitors offer advantages beyond lowering intraocular pressure; they enhance blood flow to eye tissues and protect optic nerve cells from oxidative stress, resulting in improved neuron survival and regeneration [8]. Fasudil, depicted as the hydrochloride salt in Figure 1, is a selective ROCK inhibitor that has demonstrated promising results in the treatment of glaucoma [7,9]. It works by inhibiting the ROCK pathway, which plays a crucial role in regulating the trabecular meshwork and canal of Schlemm’s aqueous humor outflow. Fasudil works by decreasing the formation of stress fibers and actomyosin contractility and reducing the expression of extracellular matrix proteins [10]. This leads to an increase in the aqueous outflow, leading to a decrease in IOP. In addition to its IOP-lowering effect, Fasudil has been shown to improve blood flow to ocular tissues and protect optic nerve cells against oxidative stress, promoting neuron survival and regeneration [11]. However, due to its hydrophilic nature (log P 0.16) and low molecular weight (MW 327.83 g/mL), attempts to formulate Fasudil using drug carriers like poly (lactic-co-glycolic acid) microspheres [10] and liposomes [12] have encountered issues, notably low entrapment efficiency and drug leakage. These limitations translate to low ocular bioavailability and pose a challenge for effective delivery of the drug to target ocular tissues. On the other hand, nanoparticles with mucoadhesive properties were found to be useful in modifying the pharmacokinetics and bioavailability of small hydrophilic molecules [13].

Nanoparticles have a large surface area-to-volume ratio, allowing for better entrapment efficiency [14]. They can prolong ocular drug residence time, sustain drug release, and potentially enhance ocular drug bioavailability [15]. However, when nanoparticles lack mucoadhesive properties, they are susceptible to rapid drainage from the ocular surface into the nasolacrimal duct, resulting in reduced effectiveness. Conversely, mucoadhesive nanoparticles for topical administration to the eyes were shown to prolong the precorneal retention of the topically applied formulation, hence enhancing drug bioavailability and reducing adverse effects associated with frequent dosing [16].

Chitosan (Cs) is one of the most abundant natural polysaccharides, primarily sourced from marine crustaceans [17]. Cs exhibits many advantages when employed in the formulation of nanoparticles for drug delivery, including biocompatibility and biodegradability. Moreover, the positive charge present on the surface of chitosan grants it mucoadhesive properties, rendering it a promising candidate for ocular drug delivery [18]. Cs nanoparticles interact with the negatively charged residues of sialic acid in the corneal and conjunctival mucosa, prolonging the drug’s presence and enhancing its bioavailability in the ocular region [19]. Additionally, Cs enhances ocular drug permeation by disrupting tight cell junctions in the corneal and conjunctival epithelial cell surfaces [20,21]. Sonaje et al.’s study strongly affirms the capability of chitosan to transiently and reversibly open these junctions, as indicated by their results [22].

Cs NPs have been used for delivering various drugs used in the treatment of eye conditions. These drugs include diclofenac [23], a non-steroidal anti-inflammatory agent; daptomycin, an anti-bacterial agent [24]; brimonidine, an anti-glaucoma agent [25]; and dexamethasone, a corticosteroid effective in managing ocular inflammatory disorders [26]. Cs is a promising drug delivery material with numerous advantages, but concerns have emerged regarding its potential ocular surface toxicity. Despite receiving approval from the US-FDA and EU for dietary and wound-dressing applications due to its biocompatibility and biodegradability, it has yet to achieve FDA approval for drug delivery applications [27]. Additionally, there have been reports indicating that cationic polymers can display toxicity due to their positive charge [28]. Therefore, one aspect of this article is to investigate chitosan’s ocular cytotoxicity to address some of these concerns.

Different methods have been reported for preparing Cs nanoparticles. These include ionic gelation, emulsion crosslinking, coacervation–precipitation, and spray drying [29]. Ionic gelation remains one of the most reported methods of preparation due to its simplicity and cost-effectiveness. It does not require the use of organic solvents, thus reducing the risk of toxicity due to residues [30]. This method is based on the electrostatic interactions that occur in the presence of oppositely charged groups; for example, those between protonated amino groups of Cs (positive charge) and negatively charged groups of polyanions, such as sodium tripolyphosphate (TPP), sodium alginate, and hyaluronic acid. Nanoparticles are produced due to the formation of crosslinks in between (intramolecular) and within (intermolecular) molecules [31].

Ocular surface absorption of topically applied drugs happens via the corneal or conjunctival/scleral routes. Elucidating the absorption pathway of a topically applied ocular drug is important to inform and guide further formulation development. To the best of our knowledge, no such studies have been conducted on Fasudil. Hence, the objectives of this study are to:

- Investigate the ocular absorption pathway of Fasudil hydrochloride using an appropriate ex vivo model.
- Develop a chitosan nanoparticulate system using the ionic gelation method and investigate (using statistical experimental design) the parameters that affect the characteristics of chitosan nanoparticles.
- Conduct in vitro release and ex vivo permeation studies of Fasudil from the prepared chitosan nanoparticles and assess their ocular (conjunctival and corneal) tolerability.
- Evaluate the cytotoxic effect of the formulated system on a human lens epithelial cell line using the neutral red uptake assay.

## 2. Materials and Methods

### 2.1. Materials

Fasudil hydrochloride was obtained from Biosynth Ltd., Compton, UK. Low-molecular-weight chitosan (deacetylation degree 75–85%) (Cs), sodium tripolyphosphate (TPP), fetal bovine serum (FBS), Trypan blue, sterile phosphate-buffered saline, Dimethyl sulfoxide (DMSO), penicillin–streptomycin, 0.25% trypsin-EDTA, and neutral red were all purchased from Sigma Aldrich Chemical Co., London, UK. Glacial acetic acid, Industrial Methylated Spirit (IMS), and absolute ethanol were purchased from Fisher Scientific, Leicestershire, UK. Eagle’s Minimum Essential Medium (EMEM) and Human Lens Epithelial Cell line (HLEC line-B3) were obtained from the American Type Culture Collection (ATCC), London, UK. All other solvents and buffer salts were obtained from Sigma Aldrich Chemical Co., London, UK; they were of analytical grade and used as received.

### 2.2. High-Performance Liquid Chromatography (HPLC) Analysis of Fasudil

Fasudil was separated and quantified by liquid chromatographic analysis using an Agilent HPLC Infinity system (Agilent, Waldbronn, Germany) coupled with a photodiode array detector (PDA). Separation was achieved using the Phenomenex^®^ Gemini 5 µm C6-Pheny column (150 × 4.6 mm) at room temperature (Phenomenex, Torrance, CA, USA). The mobile phase consisted of 90/10 (*v*/*v*) purified water/acetonitrile, with the addition of 0.1% trifluoroacetic acid by volume to both phases. Prior to use, the mobile phases were degassed using bath ultrasonication to eliminate any trapped gas bubbles. The flow rate was set at 1 mL/min, and the injection volume was 10 μL. When analyzed at a λmax of 235 nm, Fasudil exhibited a retention time of 3.5 min. The validation of the method was carried out in accordance with FDA guidelines. A calibration curve was established within concentration ranges of 2–75 μg/mL, exhibiting a coefficient of determination value of 0.999 and a curve equation (y = 40.889X + 2.5458). The method demonstrated high accuracy and selectivity, with a low limit of detection (LLOD) of 0.22 µg/mL and a low limit of quantification (LLOQ) of 0.65 µg/mL.

### 2.3. Ex Vivo Permeation of Fasudil Hydrochloride through Excised Bovine Cornea and Sclera

In this ex vivo trans-corneal/scleral permeation study, freshly excised bovine corneas and scleras were used. The experiments were conducted with a Franz diffusion cell apparatus (FDC-6, Logan Instrument Corp., Somerset, NJ, USA), which provided a diffusion area of 1.7 cm^2^. The bovine eyes were obtained from a local slaughterhouse (ABP Food Group, Guildford, UK) and transported to the laboratory in cold saline (8–10 °C). Prior to dissection, the eyes were examined for corneal damage. The excised bovine ocular membrane (cornea or sclera) separated the two compartments in such a way that the epithelial surface of the cornea/conjunctival side of the sclera faced the donor chamber. The receptor chamber (12 mL) was filled with simulated tear fluid (STF). STF was prepared by dissolving 0.67 g NaCl, 0.21 g NaHCO_3_, and 0.008 g CaCl_2_.2H_2_O in 100 mL of purified water and adjusted to pH 7.4 [32]. The temperature of the cell was maintained at 35 ± 0.5 °C with continuous stirring. Samples of the Fasudil solution (2 mL, 1 mg/mL) were placed into the donor compartment. At predetermined time intervals (every hour), a sample (0.4 mL) of the receptor medium was taken and immediately replaced with an equivalent volume of fresh STF. The quantity of Fasudil permeated was subsequently measured using HPLC. A graph was created by plotting the cumulative amount of Fasudil permeated vs. time, and this graph was then used to calculate the apparent permeability coefficient (Papp, cm/s) as follows:

$$Papp=\frac{\Delta Q}{\Delta t\left(3600\right)AC0}$$

where ΔQ/Δt indicates the permeability flux rate of Fasudil (μg/cm^2^.h) through the excised bovine corneas, calculated as the slope from the graph plotting the amount of Fasudil permeated vs. time; A is the exposed area (cm^2^) of the corneal/scleral surface; C0 represents the initial concentration (μg/mL) of Fasudil; and 3600 is the conversion constant from hours to seconds. The lag time (tL) calculation involves using the intercept and slope values from a regression line of permeation studies.

### 2.4. Statistical Experimental (Factorial) Design

A two-level full factorial design was utilized to investigate the effects of three independent variables (Cs concentration, Cs:TPP mass ratio, and sonication time) on the desired responses (dependent variables), namely, particle size, polydispersity index (PDI), zeta potential, and entrapment efficiency (EE) (Table 1). The design of 8 experimental runs and one center point was developed using Minitab software, version 21.4 (Minitab, Inc., State College, PA, USA). All experiments were carried out in triplicate (Table 2). Response surface plots were generated to demonstrate how independent variables affect the response.

### 2.5. Preparation of Chitosan Nanoparticles (Cs NPs)

Cs NPs were prepared via the ionic gelation method as previously reported by Calvo et al. [33], with minor modifications. Briefly, Cs solutions at concentrations of 1.2, 3.7, and 6.2 mg/mL were prepared by dissolving appropriate amounts of Cs (with low molecular weight and degree of deacetylation ranging between 75 and 85%) in 8 mL of acetic acid (1% *v*/*v*, pH 4). Next, 1 mL of Fasudil aqueous solution (0.5% *w*/*v*) was added to the Cs solution and stirred for 10 min. To prepare the nanoparticles, 1 mL of aqueous TPP solution (Table 2) was added dropwise to the Cs solutions by means of sonication over an ice bath using a probe sonicator (150 plus, MSE, London, UK) at 16% amplitude. The nanoparticles were separated by centrifugation at a speed of 40,000× *g* for 45 min at 4 °C. Then, the nanoparticles were washed by dispersing the formed pellet in distilled water and recentrifuged at the same previous conditions; then, the washed pellet was lyophilized, collected, and stored for further characterization.

### 2.6. Characterization of Fasudil-Cs NPs

#### 2.6.1. Particle Size, Polydispersity Index (PDI), and Zeta Potential Measurements

The particle size and PDI were analyzed at 25 °C using dynamic light scattering with a Malvern Zetasizer (Malvern Instruments Ltd., Malvern, UK). Freshly prepared Cs NP samples were used for these measurements. For particle size analysis, a square disposable cuvette was employed, and all samples were diluted with deionized water at a ratio of 1:10 (*v*/*v*). The zeta potential of the nanoparticles was also determined using the same instrument. In this analysis, a folded capillary cell was used, and all samples were diluted with deionized water at a ratio of 1:100 (*v*/*v*). Electrophoretic mobility analysis was conducted at 25 °C with a scattering angle of 90°. Each experiment was conducted in triplicate, and the results are presented as the mean ± SD. These measurements were performed with a 120 s interval between each sample run. Data were obtained by measuring the random changes in the intensity of light scattered from the moving nanoparticles.

#### 2.6.2. Scanning Electron Microscopy (SEM)

The surface morphology of the optimized Cs NPs was examined by scanning electron microscopy (SEM, Zeiss Evo50-Oxford instrument, Cambridge, UK). A drop of the nanoparticle suspension was placed over a slide cover and left to dry at room temperature before being attached to an aluminum stud using carbon double-sided tape. The samples were coated with a layer of gold using a sputter coater. Nanoparticle morphology was determined at 20.00 kV.

#### 2.6.3. Entrapment Efficiency (EE) determination

The amount of Fasudil entrapped within the nanoparticles was determined using an indirect method by calculating the amount of unentrapped drug (free drug) in the supernatant. Following the previously mentioned process, the nanosuspension was centrifuged, and the clear supernatant containing the free, unentrapped drug was carefully collected and diluted with deionized water. The amounts of Fasudil in the Cs NPs were determined using the validated HPLC method mentioned above. The percentage of drug entrapment efficiency (EE%) was then calculated by using the following equation:

$$EE\left(\%\right)=\frac{\left(Initial\;amount\;of\;drug-Free\;drug\right)}{Initial\;amount\;of\;drug}\times 100$$

### 2.7. In Vitro Drug Release Study

An in vitro release study of Fasudil from Cs NPs was conducted using a standard Franz diffusion cell (Copley scientific limited, Colwick, UK). Receptor chambers (11 mL volume) were filled with freshly prepared STF and constantly stirred using small magnetic bars. Lyophilized Cs nanoparticles (20 mg) were dispersed in two milliliters of PBS (pH 7.4) and transferred into the donor chamber before sealing it with Parafilm. The two chambers of Franz cells, a donor and a receptor, were separated by a dialysis membrane (12,000–14,000 Dalton molecular weight cut-off). The membrane was previously pre-soaked overnight in the receptor medium. The Franz cells were set at 35 ± 0.5 °C in order to mimic the ocular surface temperature. Care was taken to eliminate air bubble entrapment at the membrane/liquid interface. Samples (0.4 mL each) were withdrawn at different time intervals during the running experiment and replaced with fresh receptor medium. The samples were analyzed using the developed HPLC method to measure the amount of Fasudil released from nanoparticles. The experiments were performed in triplicate.

### 2.8. Ex Vivo Trans-Corneal Permeation of Fasudil from Fasudil-Cs NPs

A modified Franz diffusion cell apparatus (FDC-6, Logan Instruments Corp., Somerset, NJ, USA) was used for the ex vivo permeation study of Fasudil from both chitosan nanoparticles and Fasudil solution across freshly excised bovine corneas. See Section 2.3 for the detailed experimental procedure. In the donor compartment of the apparatus, 2 mL each of both the Fasudil solution and the Fasudil-Cs NPs dispersion, was placed. The samples withdrawn from the receptor compartment were analyzed using the developed HPLC method to quantify the amount of Fasudil permeated. Samples were measured in triplicate, and the results were presented as the mean value ± SD. A graph was created by plotting the amount of Fasudil that permeated vs. time. The permeation flux, apparent permeability coefficient, and lag time were calculated.

### 2.9. Conjunctival Irritation Test

The conjunctival tolerability of the Cs NPs was evaluated by a modified HET-CAM test, as reported previously [34]. Freshly collected fertilized hen’s eggs were incubated for 3 days at 37.5 ± 0.5 °C and 65 ± 5% relative humidity. During the incubation period, eggs were manually rotated 4–5 times a day and left in a horizontal position to ensure the correct development and viability of the embryo. On day three, the eggshells were sterilized with 70% IMS and opened by cracking at the edge of the growing chamber. The contents of the egg were poured into the fabricated growing chamber. Each egg was examined, and only viable embryos with intact chorioallantoic membranes (CAMs) and yolk sacs were further incubated at the same conditions after being covered with a Petri dish. On day 10 of incubation, 0.2 mL of each test formulation and control solution was gently dropped onto the surface of the CAM. Sodium hydroxide (0.5 M) was used as a strong irritant, propylene glycol as a moderate irritant, and normal saline as a negative control. After treatment, the CAMs and blood vessels, including the capillary system, were visually observed at three time points over 5 min (0.5, 2, and 5 min) to monitor the following irritant effects (responses): hyperemia, hemorrhage, and clotting. A time-dependent numerical score was assigned to every response, as indicated in Table 3. The cumulative score obtained for all three irritant responses provides a single numerical value (cumulative score). This numerical score serves as an interpretation of the conjunctival irritation potential of the respective test substance (Table 4). In the evaluation of each substance, three CAMs were employed for each test sample.

### 2.10. Bovine Corneal Opacity and Permeability (BCOP) Assay

Freshly excised bovine eyes were carefully examined for epithelial integrity and corneal damage, and those presenting defects, such as neovascularization, pigmentation, or scratches, were discarded. Plastic weighing boats were used to hold the eyes with the cornea facing upwards in the humid atmosphere of a closed shaking water bath shaken for 10 min at 37 ± 0.5 °C. Silicone O-shaped rings were placed on the center of the cornea in order to localize the effect of the testing materials. The positive and negative controls used were NaOH (0.5 M) and NaCl (0.9% *w*/*v*), respectively. A drop of saline was applied to the corneal surface before incubating for 5 min. Testing materials and controls (0.1 mL) were applied to the corneal surface for 30 s before the eyes were rinsed with 10 mL of normal saline and left in incubation for 10 min. Then, the degree of corneal damage was visually determined from the extent of opacification and was further assessed for corneal epithelium integrity using sodium fluorescein staining (2% *w*/*v*, pH 7.4) under a cobalt blue light. The observations were graded according to individual numerical scores in terms of opacity, epithelial integrity, and epithelial detachment, as reported previously [35]. The cumulative scores were calculated, and the mean of 3 independent scores was used to interpret the corneal irritation potential for the test materials (Table 5).

### 2.11. Human Lens Epithelial Cell Viability Investigation Using the Neutral Red Uptake Assay

Cell viability upon exposure to varying concentrations of Cs NPs was evaluated by providing a quantitative estimation of the number of live cells in a culture. Neutral red uptake assays were carried out on human lens epithelial cells (HLECs B-3) to assess the cytotoxicity of the studied Cs NPs. The assay depends on the ability of living cells (with functional lysosomes) to take up and bind the basic dye neutral red (which is a pH indicator), while dead cells cannot take up the dye. Liquid nitrogen-stored HLEC B-3 cells were thawed and grown in a humidified incubator at 37 °C in an atmosphere of 5% CO_2_ and 95% air. All cell lines were cultured in cell complete medium (EMEM) supplemented with 20% (*v*/*v*) fetal bovine serum and 1% (*v*/*v*) penicillin–streptomycin. Cells were passaged by trypsinization using trypsin-EDTA (0.25%) and seeded in a cell culture flask for adherent cells. A neutral red assay was conducted as previously described, with slight modifications [36,37]. In brief, cells were seeded into 96-well plates at a seeding of 8 × 10^3^ cells in a total complete medium volume of 200 µL per well and incubated at 37 °C with 5% CO_2_ for 24 h. Then, the complete medium (EMEM + 20% FBS + 1% penicillin–streptomycin) was discarded, and cells were exposed to different concentrations of Cs NPs dispersed in treatment medium (EMEM + 5% FBS + 1% penicillin–streptomycin) and incubated for 24 h. After medium aspiration, the cells were washed with 150 μL of phosphate-buffered saline (PBS) and 100 μL of complete medium supplemented with neutral red (40 μg/mL), then added to the cells and incubated for three hours at the same conditions. Later, the cells were again washed with PBS, and an NR de-staining solution (50% ethanol, 49% deionized water, and 1% glacial acetic acid) was added to the cells. The plates were shaken for 10 min to extract the NR. The optical density (OD) was measured at 540 nm using a plate reader to calculate the cell survival rate.

### 2.12. Statistical Analysis

All experiments were conducted in triplicate, and the results were presented as the mean value ± standard deviation (SD). A one-way analysis of variance (ANOVA) was performed using Minitab^®^ software, version 21.4, (Minitab, Inc, State College, PA, USA). The resulting data were deemed statistically significant when the *p*-value was less than 0.05.

## 3. Results and Discussion

### 3.1. Ex Vivo Permeation of Fasudil Hydrochloride through Excised Bovine Cornea and Sclera

**Figure 2:**
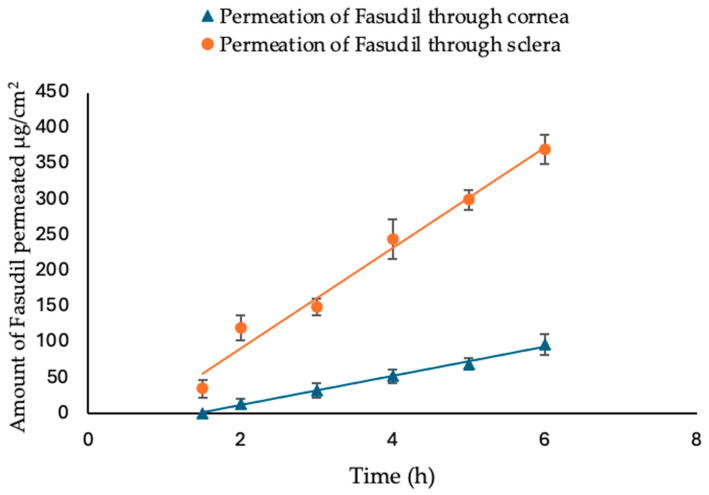
Ex vivo permeation study of Fasudil from Fasudil simple solution through excised bovine cornea and sclera for 6 h. Results are expressed (n = 3 ± SD), *t*-test was performed.

To facilitate the development of a more effective ocular formulation for Fasudil, it is vital to investigate its permeability across the primary barriers located at the front of the eye, namely the cornea and sclera. The trans-corneal/scleral permeation profiles of Fasudil solution were investigated using bovine excised eyes. Permeation parameters, including the apparent permeability coefficient (Papp), flux, and lag time, were calculated for Fasudil across both membranes (cornea and sclera). The results from trans-corneal/scleral permeation indicated that both the cornea and sclera remained intact throughout the experiment, as evidenced by the linear permeation profile (Figure 2). The amounts of Fasudil that permeated at 2 h and 6 h were calculated and used to compare and contrast the resulting profiles. The amount of Fasudil that permeated through the cornea at 2 h and 6 h was 14.4 µm/cm^2^ and 96.8 µm/cm^2^, respectively. Fasudil permeation through the sclera was significantly higher (*p* < 0.05), with 109.1 µm/cm^2^ and 370.0 µg/cm^2^ at the same time points, respectively.

Table 6 provides a summary of the trans-corneal/scleral permeation parameters for the Fasudil solution. Both the steady flux and Papp values for Fasudil permeation through the cornea were significantly lower (*p* < 0.05) compared to those observed for the sclera. These results suggest that Fasudil permeates at a higher rate through the sclera when compared to the cornea (approximately 3.4 times higher). This difference can be attributed to the distinct structural characteristics of these eye tissues. The sclera, characterized by collagen-based pores, facilitates easier permeation of hydrophilic molecules [38]. In contrast, the hydrophobic nature of the external corneal stratified epithelium acts as a barrier, impeding the permeation of hydrophilic substances [38].

The permeation data generated from this experiment clearly highlight the need for a formulation strategy to perturb and overcome the barrier properties of the cornea in order to improve the ocular absorption of Fasudil. Chitosan nanoparticles are likely to achieve such an objective.

### 3.2. Design of Experiment

Design of experiment (DoE) was performed to optimize Cs nanoparticles and investigate the correlation between responses and factors. Independent factors were Cs concentration, Cs:TPP mass ratio, and sonication time. The data generated by the Minitab software system, version 21.4, were employed to evaluate the effects of independent factors and their interaction effects on particle size, PDI, zeta potential, and EE. The design of eight experimental runs was developed in order to optimize the properties of nanoparticles produced in terms of particle size (less than 300 nm), high surface charge, and entrapment efficiency. ANOVA was used to check the significance of the effect on the responses when the *p*-value was < 0.05. Table 7 presents the outcomes related to particle size, PDI, zeta potential, and entrapment efficiency for the generated formulations.

### 3.3. Characterization of Fasudil-Cs NPs

#### 3.3.1. Effect of Independent Variables on Particle Size and PDI

**Figure 3:**
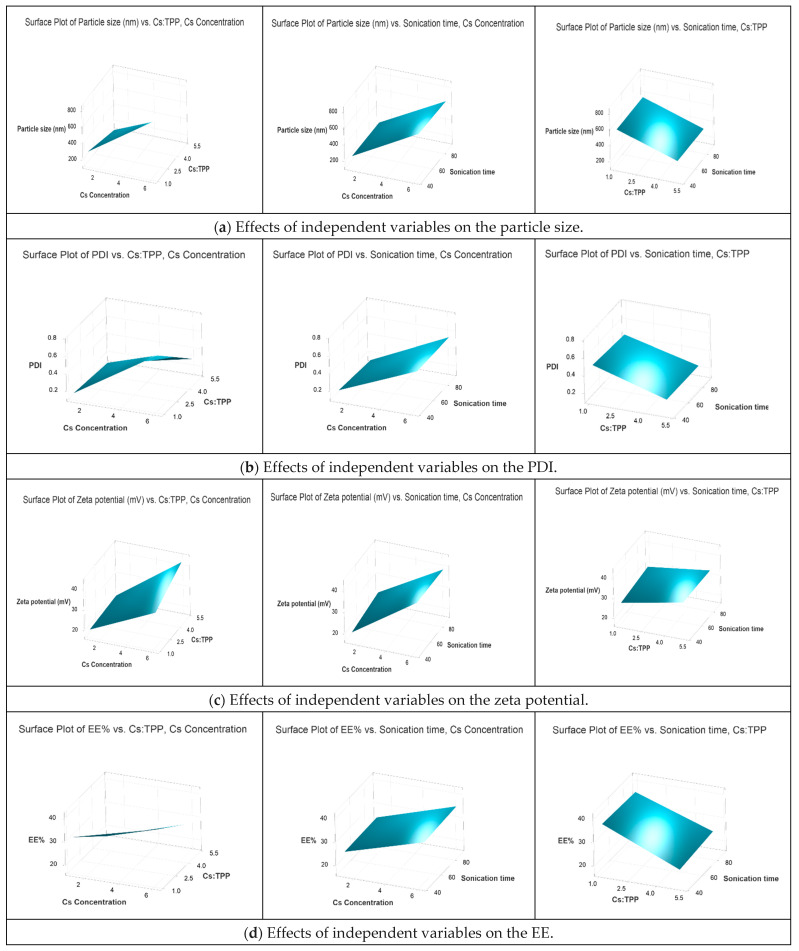
Response surface representing the effect of independent variables, including Cs concentration (mg/mL), Cs:TPP mass ratio, and sonication time (s), on the dependent variables: (**a**) particle size (nm), (**b**) PDI, (**c**) zeta potential (mV), and (**d**) entrapment efficiency (EE%).

Particle size and PDI are essential factors, considering their importance for effective topical ophthalmic administration. A small particle with a more uniform size provides higher ocular bioavailability by increasing permeation across ocular tissues and is less irritating [39,40]. The main effects of the investigated independent variables and their interactions on the particle size were observed in a 3D surface plot (Figure 3a). The results indicate that Cs concentration, Cs:TPP mass ratio, and their interaction significantly impacted particle size. An increase in the concentration of Cs led to the creation of large-sized nanoparticles, which agrees with previous reports [41,42,43]. When the concentration of Cs rises, the molecules become more interlinked, and viscosity increases, making it difficult for TPP anions to disperse within the Cs molecules. This leads to ineffective crosslinking, which results in the formation of larger particles [44]. The creation of Confirmed, ‘n’ should not appear in italics as variables. nanoparticles mainly depends on the ionic interactions between the molecules of Cs and TPP. Therefore, it is believed that the mass ratio between the two molecules has a crucial role in defining the size of the nanoparticles [33,45]. When the mass ratio of Cs to TPP was changed from 5:1 to 1:1, the size of the nanoparticles was significantly increased (*p* < 0.05). As the Cs TPP mass ratio decreased, more sodium tripolyphosphate became accessible. Hence, excess sodium tripolyphosphate linked the mono-nanoparticles to produce larger nanoparticles. Similar behavior was also seen at lower Cs:TPP mass ratios by Hu et al. [45] and Antoniou et al. [46]. These findings were consistent with the data provided by Leelapornpisid et al. [47], who claimed that maintaining Cs and TPP concentrations within a certain range is necessary for producing Cs NPs on a nanoscale. In terms of polydispersity index (PDI), the results showed that the pattern of particle size was linearly associated with the PDI, i.e., an increase in particle size was accompanied by an increase in PDI (Figure 3b).

#### 3.3.2. Effect of Independent Variables on Zeta Potential

The positive values of the zeta potential are due to the presence of protonated Cs amino groups on the surface of the nanoparticles [48,49]. The 3D surface plot in Figure 3c demonstrates a significant effect of Cs concentration on the nanoparticle’s zeta potential. The zeta potential increased with increasing Cs concentration due to the increased amount of protonated amino groups of Cs in the system [50,51,52,53]. The given data imply a positive correlation between the zeta potential and the Cs:TPP mass ratio. The surface charge dropped when the Cs-to-TPP mass ratio decreased from 5:1 to 1:1. The reduction in zeta potential at lower Cs:TPP mass ratios may result from the neutralization of positively charged amino groups on Cs by TPP anions [50,54]. However, the ANOVA results showed that Cs concentrations had the most significant impact on the zeta potential of the produced nanoparticles, while the sonication time had no effect.

#### 3.3.3. Effect of Independent Variables on EE

The results obtained from measuring the amount of Fasudil in the supernatant after centrifugation of the nanoparticles indicated an EE range of 17.2–41.40%. The relationship between Cs concentration and EE is positive. This means that increasing the concentration of chitosan leads to an increase in entrapment efficiency, as can be observed in Figure 3d. When the Cs concentration was increased, the protonated amino group was more easily accessible in the system. As a result, the crosslinker, TPP, had access to additional binding sites, which increased the EE% [51,52,53]. However, the Cs:TPP mass ratio had the opposite impact on the EE%. As the mass ratio of Cs to TPP increased, the EE% decreased. This could be explained by the limited quantity of TPP anions available for crosslinking with Cs. Similar results were reported by Abosabaa et al. [53]. However, the relatively low EE of Fasudil in the nanoparticles could be attributed to the hydrophilic nature of the drug by causing partitioning into the hydrophilic phase rather than trapping in the generated nanoparticles. These findings were also supported by Lazaridou et al., who claimed low EE in Cs TPP nanoparticles because of their high water solubility [55].

The application of the desired constraints, such as the maximum EE% and a particle size of less than 300 nm, led to formulation optimization. With a computed desirability of 0.76, a set of conditions was identified and chosen for further formulation development, namely, a Cs concentration of 1.2 mg/mL, a Cs:TPP mass ratio of 1:1, and a sonication time of 45 s.

#### 3.3.4. Scanning Electron Microscopy (SEM)

**Figure 4:**
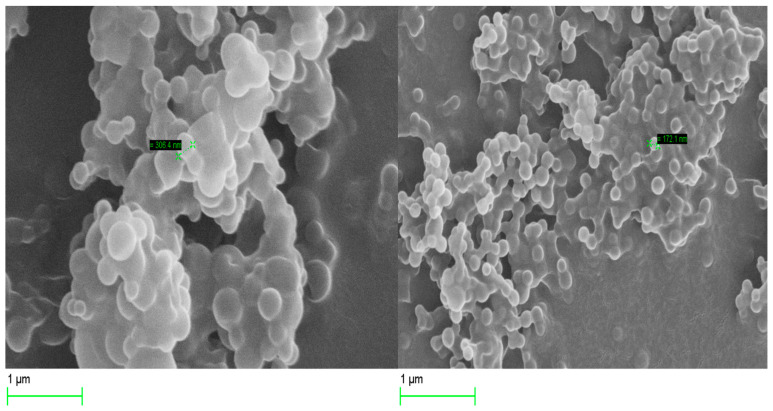
Scanning electron micrographs of optimized Cs nanoparticles loaded with Fasudil hydrochloride.

Figure 4 shows SEM images of the optimized Cs NPs loaded with Fasudil. The particles exhibit typical spherical shapes with evidence of particle aggregation. It is important to point out that the observed particle aggregation could be a consequence of the drying process during SEM sample preparation [56,57].

### 3.4. In Vitro Drug Release Study

**Figure 5:**
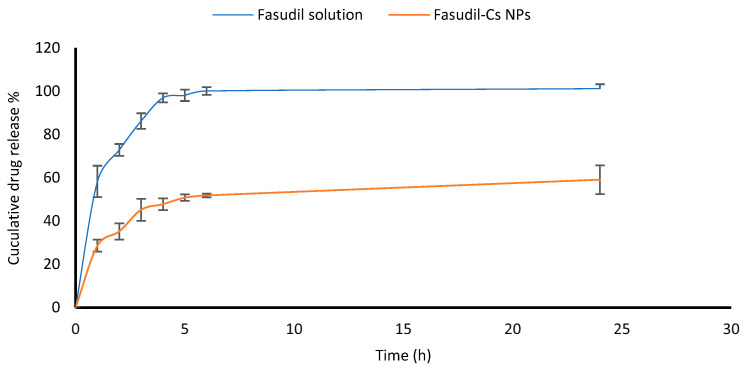
Cumulative release of Fasudil loaded into Cs NPs and Fasudil solution (control) in simulated tear fluid (STF). Results are presented as mean ± SD, n = 3.

The ability of Cs NPs to release the drug in vitro was investigated in STF under physiological conditions representative of the ocular surface (pH 7.4, 35 °C) for 24 h. The performance of the optimized chitosan nanoparticles was compared to that of the Fasudil solution. Figure 5 shows that approximately 60% of the drug was recovered in the receptor chamber from the solution within the first hour, indicating that the drug had the ability to permeate almost unrestrictedly through the dialysis membrane. In contrast, Cs NPs displayed an initial burst release of Fasudil followed by a sustained release over 24 h. An initial burst release of 35.51% occurred within two hours, followed by an extended release of 47.8% and 55.6% at four and six hours, respectively. The initial burst release may be associated with the desorption of the Fasudil molecules that have been adsorbed at the surface of the Cs nanoparticles or those poorly trapped within the polymeric matrix. From a clinical point of view, an initial rapid release may be advantageous to achieve the therapeutic concentration of the drug in the shortest amount of time, followed by a sustained release. The drug’s presence in the particle’s core may be responsible for the sustained release. M. Bin-Jumah et al. reported similar release profiles for clarithromycin, an anti-bacterial agent [48].

The release kinetics of Fasudil from chitosan nanoparticles were assessed using the zero-order, first-order, Higuchi, and Korsmeyer–Peppas release models. Table 8 displays the generated coefficient of determination (R^2^) values derived from the model fitting results of the release data. The values reveal the best fit of the release data with the Higuchi and Korsmeyer–Peppas models (Table 8), suggesting that the release of Fasudil is primarily governed by diffusion. Additionally, the release exponent (n) value obtained from the Korsmeyer–Peppas model is 0.35, confirming that Fickian diffusion serves as the predominant process influencing drug release.

### 3.5. Ex Vivo Trans-Corneal Permeation of Fasudil from Fasudil-Cs NPs

**Figure 6:**
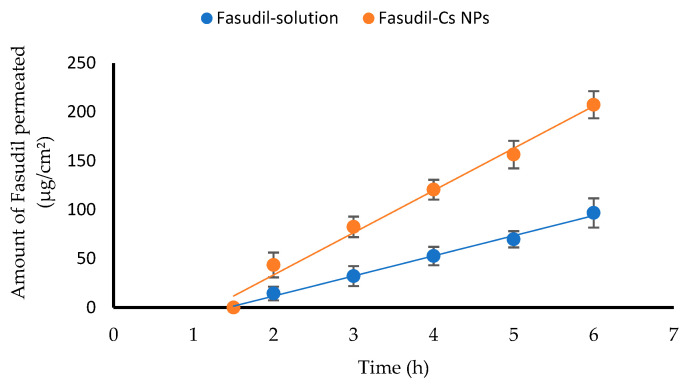
Trans-corneal permeation profiles of Fasudil from Cs NPs and Fasudil solution (mean ± SD, n = 3).

Figure 6 illustrates the trans-corneal permeation profiles of Fasudil through excised bovine corneas. Permeation parameters such as the apparent permeability coefficient (Papp), flux, and lag time (tL) were calculated. Notably, Cs NPs demonstrated a substantial enhancement in Fasudil permeation through the cornea compared to the Fasudil aqueous solution. The flux and Papp values for Cs NPs were 2.1-fold higher than those for the Fasudil solution (Table 9). The cornea’s ability to effectively control Fasudil permeation is evidenced by the linearity of the Fasudil permeation profile. The significant improvement in permeation (*p* < 0.05) can be attributed to the ionic interactions between the negatively charged corneal surface and the positively charged amino groups on Cs NPs. These findings align with a previous report [58]. Additionally, increased permeation might be caused by the ability of Cs nanoparticles to transiently affect the barrier properties between corneal epithelial cells, which could potentially increase drug flux through the cornea [59].

Reflecting on both in vitro release and ex vivo permeation results of Fasudil from Cs NPs and extrapolating these findings to what is likely to happen once applied to the ocular surface, one could postulate that a faster (pulsed) release along with poor corneal permeation from the aqueous solution would result in quick ocular surface loss, leading to poor corneal absorption. On the other hand, a slower Fasudil release along with improved corneal permeation (characteristics of Cs NPs) is likely to result in improved corneal absorption.

### 3.6. Conjunctival Irritation Test

**Figure 7:**
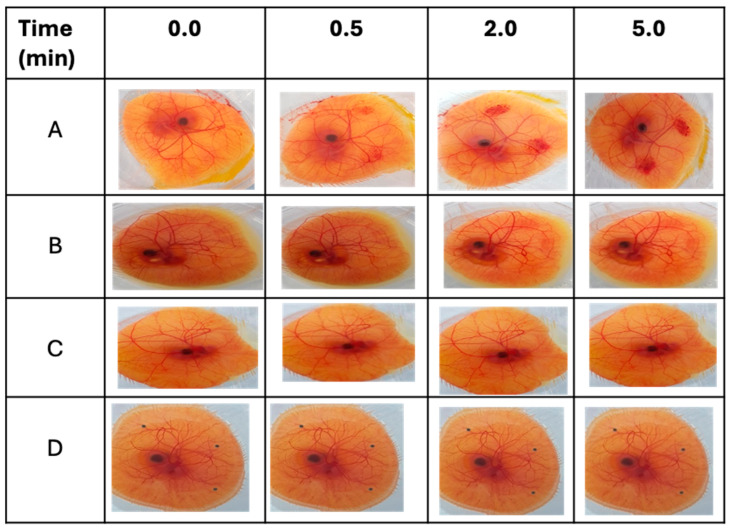
Images displaying the irritant effects of substances applied to the CAMs over a 5 min period: (A) NaOH 0.5 M (strong irritant), (B) propylene glycol (PG) (moderate irritant), (C) normal saline (negative control), and (D) Cs NPs formulation.

**Figure 8:**
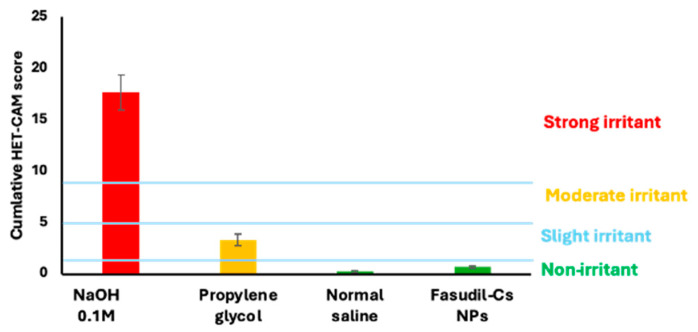
Cumulative scores of the HET-CAM assay results and subsequent classification for the controls and Cs NPs. Results are expressed as mean values ± SD, n = 3.

The HET-CAM assay is beneficial for assessing the irritation effect of test substances on ophthalmic tissues such as the conjunctiva [60]. This is because the CAM is a functional membrane that encompasses vasculature and inflammatory responses that can be analyzed for ocular injury-related outcomes. The HET-CAM assay measures the capacity of a test substance to harm blood vessels and provoke adverse effects like hyperemia, hemorrhage, and clotting/coagulation [61]. As seen in Figure 7A, a strong irritant reaction, including clotting and hemorrhage, occurred when the CAM was subjected to a 0.5 M NaOH solution, a potent irritant. Testing with a moderate irritant (propylene glycol) produced a weaker response (compared with NaOH), as depicted in Figure 7B. This finding aligns with the previous report by Smail et al., which stated that propylene glycol can cause a minor irritant effect when used on its own [34]. On the other hand, neither the Cs NPs as depicted in Figure 7D nor the saline solution (negative control) produced any signs of ocular irritation (Figure 7C). The results suggested that the formulated Cs NPs are non-irritant when applied to the surface of the CAM. Figure 8 shows the cumulative scores of the controls and Cs NPs.

### 3.7. Bovine Corneal Opacity and Permeability (BCOP) Assay

**Figure 9:**
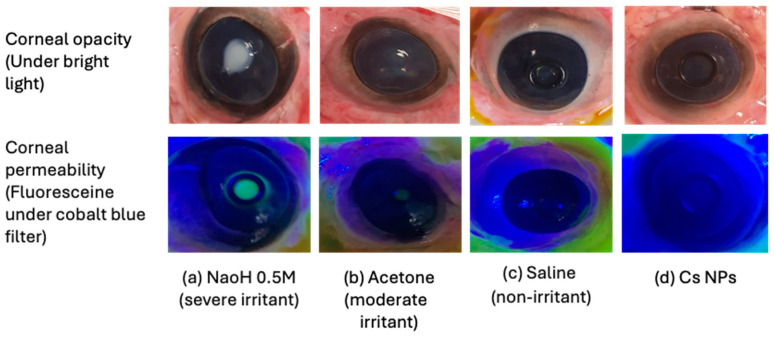
Bovine corneal opacity (upper row) and permeability assay (lower row). The corneas were subjected to controls (**a**–**c**) and Cs NPs (**d**) and examined for opacity and permeability under bright light and a cobalt blue filter, respectively.

The BCOP is an ex vivo assay that determines how topically applied substances alter the opacity and permeability of freshly excised bovine corneas [34]. Corneal opacity is generally caused by protein denaturation or precipitation in the epithelial or stromal layers as a result of exposure to an irritant [62]. The permeability of the cornea is assessed using sodium fluorescein, a dye that normally cannot pass through corneal epithelial cells. Permeability may rise in the presence of injury to the corneal epithelium [34]. Images of the bovine’s corneas before and after staining are shown in Figure 9. Corneal opacity and fluorescein staining could be seen in images of the positive control, 0.5 M NaOH, a severe irritant (Figure 9a). The use of NaOH led to denaturation of the corneal proteins, resulting in a reduction in corneal transparency and an increase in corneal opacity. The damaged epithelium layer allowed for greater penetration of the fluorescent dye into the stroma, which was identified through fluorescent staining and reduced corneal luster. Acetone had a less strong irritating effect on the cornea, resulting in slight opacity and weak fluorescein staining (Figure 9b). However, no signs of corneal opacity or fluorescein staining were observed with either the negative control (where the corneas were treated with a normal saline solution) or the Cs NP formulation, as depicted in Figure 9c and Figure 9d, respectively.

**Figure 10:**
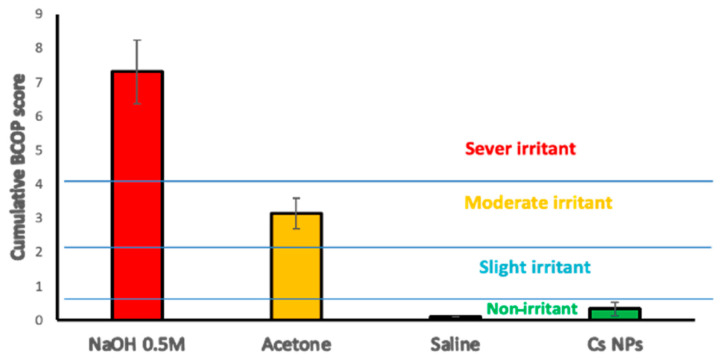
Cumulative scores of the BCOP assay and subsequent irritation classification for the controls and Cs NPs. Results are expressed as mean values ± SD, n = 3.

The BCOP scores for corneal opacity and epithelial integrity were plotted (Figure 10) for severe, moderate, and non-irritant controls, as well as Cs NPs. The cumulative score for Cs NPs was consistently below 0.5, suggesting that it has a low-to-negligible potential for causing corneal irritation.

### 3.8. Human Lens Epithelial Cell Viability Investigation Using the Neutral Red Uptake Assay

**Figure 11:**
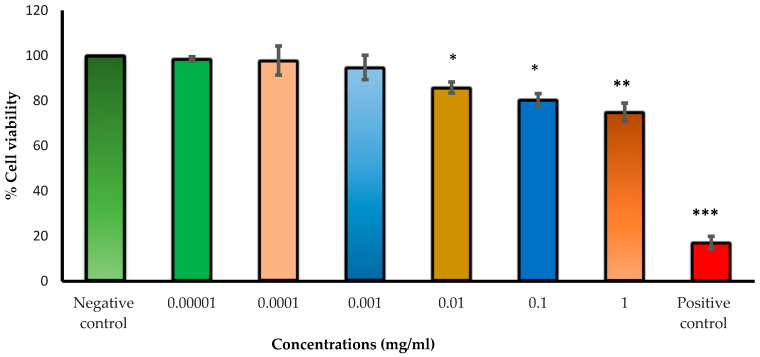
Cell viability (%) evaluated by NRU assay after 24 h of exposure of HLEC cells to different concentrations of Cs NPs. Hydrogen peroxide and treatment medium are used as positive and negative controls, respectively. Results are expressed as mean values ± SD from three independent experiments. One-way analysis of variance (ANOVA) followed by Bonferroni post hoc test was used with * *p* < 0.05, ** *p* < 0.01, and *** *p* < 0.001.

The neutral red uptake assay is commonly used for measuring cytotoxicity; it provides a quantitative assessment of the number of viable cells present in a culture [63]. It is based on the ability of cells to incorporate and bind the neutral red dye in lysosomes, which is later extracted [64]. The dye’s net charge (near zero) at physiological pH allows it to penetrate cell membranes. Within the lysosomes, there is a proton gradient to keep the pH lower than in the cytoplasm. The neutral red dye becomes charged and is hence retained in the lysosomes. However, if the cell dies or the pH gradient decreases, the dye is released [63]. In this study, the cytotoxicity of Cs NPs was assessed by subjecting HLEC cells to different concentrations of the nanoparticles (ranging from 1 × 10^−5^ to 1 mg/mL) using the NRU assay. Cell viability was determined by comparing the obtained results to those of the negative control (treatment medium), as shown in Figure 11. Hydrogen peroxide was employed as a positive control, and it yielded a considerably low cell viability of 17%, reflecting its cytotoxic effects. Hydrogen peroxide is an oxidizing agent that can damage cells and cause their death. The cells showed great tolerance to low concentrations of Cs NPs (ranging from 1 × 10^−5^ to 1 × 10^−3^ mg/mL), as evidenced by a cell viability of over 94%. However, when the concentration was increased to 1 mg/mL, cell viability decreased significantly to 75% (*p* < 0.05). This is in line with previous studies indicating that Cs NPs exhibit concentration-dependent cytotoxicity, where higher concentrations lead to more severe cytotoxicity [65].

## 4. Conclusions

In conclusion, we observed significant differences in Fasudil permeation between the cornea and sclera during ex vivo studies. The tendency of the cornea to act as a barrier to the absorption of Fasudil warranted our move to use chitosan nanoparticles, as they are known to increase corneal permeability. The size and Fasudil entrapment efficiency of chitosan nanoparticles were found to be highly influenced by the quantity of chitosan used. Additionally, our study demonstrated that chitosan nanoparticles enabled a controlled release of Fasudil, highlighting their potential for sustained drug delivery with less frequent topical administration compared to an aqueous solution. Importantly, our study demonstrated the conjunctival and corneal biocompatible nature of these nanoparticles. Moreover, the excellent tolerance of lens epithelial cells to low concentrations of chitosan nanoparticles further demonstrates their ocular biocompatibility. These findings collectively support the promise of Fasudil–chitosan nanoparticles as a more efficient alternative to conventional simple solution eye drops for glaucoma therapy.

## Figures and Tables

**Table 1:** Independent and dependent variables and their levels used in the experimental design study.

Independent Variables	Low Level	High Level	Dependent Variables
Cs conc. (mg/mL)	1.2	6.2	Particle size (nm)
Cs:TPP mass ratio	1:1	5:1	Polydispersity index
Sonication time (s)	45	90	Zeta potential (mV)
			Entrapment efficiency (EE%)

**Table 2:** Matrix of 2^3^ factorial design used to optimize Cs nanoparticles.

Formulation Code	StdOrder	RunOrder	Cs Concentration (mg/mL)	Cs:TPP Mass (mg) Ratio	Sonication Time (s)
F1	3	1	1.2	5:1	45
F2	2	2	6.2	1:1	45
F3	5	3	1.2	1:1	90
F4	8	4	6.2	5:1	90
F5 *	9	5	3.7	3	67.5
F6	6	6	6.2	1:1	90
F7	4	7	6.2	5:1	45
F8	1	8	1.2	1:1	45
F9	7	5	1.2	5:1	90

* Center point.

**Table 3:** HET-CAM test scoring system.

Title	Score		
Response/Time (min)	0.5	2	5
Hyperemia	5	3	1
Hemorrhage	7	5	3
Clotting/coagulation	9	7	5

**Table 4:** Cumulative score classification of the HET-CAM.

Cumulative Score	Interpretation
0.0–0.9	Non-irritant
1.0–4.9	Slight irritant
5.0–8.9	Moderate irritant
9.0–21	Severe irritant

**Table 5:** BCOP scoring system and interpretation.

Opacity	Score	Epithelial Integrity	Score	Epithelial Detachment	Score	Cumulative Score	Interpretation
None	0	None	0	No gross abnormalities	0	≤0.5	Non-irritant
Slight	1	Diffuse and weak	0.5	Wrinkling of corneal surface	2	0.6–1.9	Slight irritant
Market	2	Confluent and weak	1	Loosening of epithelium	3	2.0–4.0	Moderate irritant
Severe	3	Confluent and intense	1.5	Epithelium absent	4	>4	Severe irritant
Opaque	4						

**Table 6:** Flux, apparent permeability coefficient (Papp), lag time (tL), and regression coefficient (R^2^) of Fasudil simple solution through cornea and sclera.

Ocular Tissues	Flux (μg/cm^2^.h)	Papp × 10^−6^ (cm/s)	Lag Time (h)	R^2^
Cornea	20.6 * ± 2.24	3.4 * ± 0.37	1.4 ± 0.17	0.9870
Sclera	70.4 ± 3.93	11.5 ± 0.64	0.7 ± 0.16	0.9745

Note: * *p* < 0.05.

**Table 7:** Particle size, polydispersity index, zeta potential, and entrapment efficiency for the generated formulations (mean ± SD, n = 3).

Formulation Code	Particle Size (nm)	Polydispersity Index	Zeta Potential (mV)	Entrapment Efficiency (%)
F_1_	149.2 ± 15.4	0.135 ± 0.008	20.8 ± 2.9	17.2 ± 2.79
F_2_	835.9 ± 23.4	0.762 ± 0.013	34.9 ± 4.0	41.4 ± 4.15
F_3_	290.9 ± 12.3	0.129 ± 0.006	22.7 ± 3.3	31.4 ± 2.53
F_4_	475.2 ± 11.8	0.334 ± 0.008	43.8 ± 3.2	30.4 ± 3.83
F_5_ *	170.8 ± 4.8	0.357 ± 0.02	16.4 ± 0.9	33.3 ± 1.5
F_6_	820.2 ± 27.4	0.756 ± 0.012	34.5 ± 2.5	40.3 ± 2.6
F_7_	492.1 ± 17.8	0.330 ± 0.037	43.8 ± 1.8	27.6 ± 3.7
F_8_	297.0 ± 13.4	0.21 ± 0.034	17.6 ± 2.3	32.0 ± 3.2
F9	144.0 ± 15.6	0.127 ± 0.007	21.4 ± 3.1	18.1 ± 3.7

* Represents the center point.

**Table 8:** The coefficients of determination (R^2^) and the release exponent (n) were obtained through model fitting of the release data of Fasudil from the optimized Cs NPs (results represent mean values ± SD, n = 3).

Coefficient of Determination	Zero Order	First Order	Higuchi	Korsmeyer–Peppas	Release Exponent (n)
* R * ^ 2 ^	0.8962 ± 0.05	0.8642 ± 0.04	0.9460 ± 0.04	0.9650 ± 0.02	0.35 ± 0.03

**Table 9:** Flux, apparent permeability coefficient (Papp), lag time (tL), and regression coefficient (R^2^) of Fasudil through cornea from Fasudil simple solution and Fasudil-Cs NPs.

Samples Title	Flux (μg/cm^2^.h)	Papp × 10^6^ (cm/s)	Lag Time (h)	R^2^
Fasudil solution	20.6 * ± 2.24	3.46 * ± 0.37	1.4 ± 0.17	0.9870
Fasudil-Cs NPs	43.2 ± 1.75	7.1 ± 0.31	1.2 ± 0.05	0.9772

Note: * < 0.05.

## Data Availability

The data presented in this study are available in this article.
